# Mechanisms of action underlying *Shentong Zhuyu* decoction based treatment of rheumatoid arthritis using systems biology and computer-aided drug design

**DOI:** 10.1097/MD.0000000000036287

**Published:** 2023-11-24

**Authors:** Shujun Bai, Xue Han, Yanchen Lan, Haodong Wang, Rui Wang, Liyuan Li, Qiuhang Song, Aiying Li

**Affiliations:** a College of Basic Medicine, Hebei University of Chinese Medicine, Shijiazhuang, China; b Hebei Key Laboratory of Chinese Medicine Research on Cardio-Cerebrovascular Disease, Hebei University of Chinese Medicine, Shijiazhuang, China; c Hebei Higher Education Institute Applied Technology Research Center on TCM Formula Preparation, Shijiazhuang, China.

**Keywords:** immune network targets, interleukin 6, system biology, Traditional Chinese Medicine

## Abstract

Rheumatoid arthritis is an autoimmune disease characterized by chronic polyarticular pain, for which no cure currently exists. In Chinese medicine, rheumatoid arthritis (RA) is believed to be caused by phlegm and blood stagnation. *Shentong Zhuyu* decoction can be used to treat RA, as it promotes blood circulation, resolves blood stasis, and relieves pain. In our study, we used network pharmacology and computer-aided drug design to evaluate the components, active compounds, and targets of Shentong Zhuyu decoction (STZY). Our results suggest that STZY contains active compounds such as quercetin, luteolin, and formononetin that regulate immune network targets. RA associated genes are enriched in pathways including those associated with nuclear factor kappa B, phosphatidylinositol-3-kinase/AKT, and hypoxia inducible factor 1 signaling. The main active compounds in STZY (quercetin and luteolin) were derived from *Achyranthis Bidentatae Radix, Carthami Flos, licorice, Cyperi Rhizoma*, and *Myrrha* and targeted the pro-inflammatory cytokines interleukin 2, interleukin 1 alpha, interleukin 1 beta, and interleukin 6. In addition, the compounds quercetin, luteolin, and formononetin in these herbs can target the anti-inflammatory cytokines interleukin 4 and interleukin 10. Our results suggest that STZY can balance the immune network, promote an anti-inflammatory environment, and reduce the clinical symptoms of RA. Based on the close relationship between inflammatory response and osteoclast formation, we hypothesized that STZY may inhibit inflammation and alleviate bone destruction in RA. Our findings indicate that STZY can treat RA through multiple components, targets, and pathways. This study may provide a reference for the clinical application of STZY in RA treatment.

## 1. Introduction

Rheumatoid arthritis (RA) is an autoimmune condition characterized by chronic polyarticular symptoms that are symmetrical and aggressive, with peripheral joints being the most severely affected. Moreover, RA causes synovial inflammation, lymphocytic infiltration, formation and proliferation of vascular opacities, erosion of articular cartilage and bone, skin ulcers, neuropathy, and organ damage. Inflammation, bone destruction, and synovial hyperplasia are recognized as the primary pathological features of RA. Under healthy conditions, a dynamic balance exists between pro-inflammatory and anti-inflammatory factors; however, if this balance is disrupted, RA may be triggered and eventually lead to bone destruction. Unfortunately, no cure for RA is currently available.^[1]^ nonsteroidal anti-inflammatory drugs (NSAIDs) are often used as traditional medicines for the treatment of RA. These NSAIDs can be used in combination with glucocorticoids and phytomedicinal agents to improve patient response. In addition, disease-modifying drugs such as methotrexate and leflunomide have been shown to have significant efficacy in controlling or delaying rheumatoid arthritis disease.^[2]^ In recent years, targeted treatments such as biological immunomodulators, including tumor necrosis factor-inhibitors (golimumab), interleukin 1 antagonists (anakinra), interleukin 6 antagonists (sirukumab), and immune therapy have been introduced.^[3]^ However, all of these commercially available drugs have serious adverse effects, such as an increased risk of respiratory infections and gastrointestinal and cardiovascular diseases, and their specific mechanisms remain unknown. Thus, new safe and effective drugs for RA need to be developed.

Continued advances in the research of traditional Chinese medicine (TCM) and its effects on various conditions, including RA, have increased our understanding of its efficacy and applicability. Multiple studies have suggested that TCM has flexibility in treating RA, fewer toxic side effects, good long-term efficacy, and strong anti-inflammatory outcomes. Within TCM, RA falls under the category of paralysis, which includes conditions induced by wind, cold, or dampness exposure, as documented in The Yellow Emperor’s Inner Classic·on Febrile Diseases. TCM attributes the cause of RA to phlegm and blood stagnation induced by an insufficiency of visceral qi and the invasion of exogenous pathogens or factors. Several common TCM remedies are used in RA treatment, including *Shentong Zhuyu* decoction (STZY), *Guizhi Fuzi* decoction, *Baishao Guizhi* decoction, *Fangji Huangqi* decoction, *Duhuo Jisheng* decoction, and *Baishu Fuzi* decoction. Most of these formulations are known for their very good efficacy in the clinical treatment of RA. In addition, in Correction on the Errors of Medical Work, STZY is described as particularly effective in the treatment of RA, as it clearly promotes blood circulation, reduces blood stasis, relieves pain, and dispels rheumatism. Wang L et al^[4]^ confirmed the feasibility of STZY for the treatment of RA by the experimental method of establishing an animal experimental model of RA. In addition, health products such as Shentong Zhuyu Pills are already on sale in the Chinese market (Production License No. SC10734160230673). The sovereign drugs in this formula, namely *Carthami Flos, Persicae Semen, Chuanxiong Rhizoma*, and *Angelicae Sinensis Radix*, have been reported to invigorate blood circulation and resolve blood stasis. The minister drugs, such as *Notopterygii Rhizoma Et Radix, Gentianae Macrophyllae Radix, Faeces Trogopterpri, Myrrha*, and *Cyperi Rhizoma*, are known to invigorate circulation, regulate qi, and relieve pain. In addition, the adjuvants *Achyranthis Bidentatae Radix* and *Pheretima* dispel wind and activate the network, thereby reducing the primary symptoms of RA. Finally, the messenger drug, *Glycyrrhizae Radix Et Rhizome*, is added to help induce the meridians, which invigorates the spleen and harmonizes the activity of the other components within this decoction. Therefore, plant-origin medicine based on STZY mining for the treatment of RA can provide new ideas for the clinical treatment of RA.

In this study, we investigated the molecular mechanism underlying STZY-mediated therapy of RA. We examined the RA-related and STZY target genes likely to be involved in these responses and identified 116 common genes. Further evaluation revealed that these target genes are enriched in several signaling pathways, including nuclear factor kappa B, phosphatidylinositol-3-kinase/AKT (PI3K/AKT), and hypoxia inducible factor 1, which are closely associated with inflammation. In addition, our evaluation evidenced that quercetin and luteolin can target disease-related genes interleukin 1 alpha (IL-1α), interleukin 1 beta (IL-1β), interleukin 2 (IL-2), and interleukin 6 (IL-6) in the pro-inflammatory factor network. Quercetin, luteolin and formononetin can also target disease targets interleukin 4 and interleukin 10 in the anti-inflammatory factor network, thus balancing the role of immune network targets in the treatment of RA. Therefore, we believe that these findings may provide a potential reference for the clinical application of STZY and theoretical support for the development of more effective drugs for the treatment of RA.

## 2. Materials and Methods

### 2.1. Active ingredients and *Shentong Zhuyu* decoction targets

We identified the channel tropism for each of the ingredients of STZY (*Chuanxiong Rhizoma* 6g, *Angelicae Sinensis Radix* 9g, *Glycyrrhizae Radix Et Rhizome* 6g, *Carthami Flos* 9g*, Myrrha* 6g, *Achyranthis Bidentatae Radix* 9g, *Notopterygii Rhizoma Et Radix* 3g, *Gentianae Macrophyllae Radix* 3g, *Persicae Semen* 9g, *Cyperi Rhizoma* 3g, *Pheretima* 6g, and *Faeces Trogopterpri* 6g)^[5]^ using the Integrative Pharmacology-based Network Computational Research Platform of Traditional Chinese Medicine (TCMIP) database,^[6]^ and the findings were visualized using BioRender (https://biorender.com/). Traditional Chinese Medicine Systems Pharmacology Database and Analysis Platform^[7]^ and TCMIP databases were used to screen potential herbal compounds and obtain their relevant target genes using bioavailability ≥ 30% and drug-likeness ≥ 0.18^[8]^ as filters for all ingredients in STZY except for flying squirrel droppings and earthworm. The chemical composition of earthworm^[9]^ and flying squirrel droppings^[10]^ and their target genes were identified by reviewing the relevant literature. These predictions were then refined and supplemented based on published literature, allowing full identification of all bioactive ingredients and corresponding target genes of STZY.

### 2.2. Genetic screening for rheumatoid arthritis-related targets

We identified disease-associated potential target genes by searching the Gene Cards (https://www.genecards.org),^[11]^ Drug Bank (https://go.drugbank.com),^[12]^ online mendelian inheritance in man (http://www.omim.org),^[13]^ therapeutic target database (https://db.idrblab.net/ttd/),^[14]^ and Pharm GKB (https://www.pharmgkb.org/)^[15]^ databases using “Rheumatoid Arthritis” as the keyword.

### 2.3. “Active ingredient-target gene” network construction

We coupled these 2 datasets in Cytoscape 3.7.2 (https://cytoscape.org/)^[16]^ and produced our first draft of an STZY “active ingredient-target gene” network. Here, the target genes and ingredients were represented as “nodes” and “spokes” were used to represent their interrelationships. Then, we analyzed the network characteristics parameters using the built-in Network Analyzer and selected the data with the highest node degree values for analysis. These evaluations allowed us to investigate the most important components and target genes in STZY as well as the relationships between them.

### 2.4. Enrichment analysis and acquisition of critical molecular complex detection (MCODE) networks

Gene ontology (GO) and Kyoto encyclopedia of genes and genomes (KEGG) Pathway enrichment analyses (*P* < .01) of each drug-disease target gene were completed via the Metascape (https://metascape.org) website,^[17]^ and key MCODE networks were obtained using MCODE algorithm on the site. In addition, the top 5 pathways ranked by biological process, cellular component, and molecular function were selected for GO enrichment mapping and the top 20 signaling pathways were selected for pathway enrichment mapping. Disease ontology (DO) enrichment analysis was performed on these key gene targets (*P* < .01) using KOBAS 3.0 (http://kobas.cbi.pku.edu.cn),^[18]^ and disease enrichment maps were plotted.

### 2.5. Establishment of immune network targets and prediction of compound potency

Based on the results of enrichment and key MCODE networks, we preliminarily identified IL family genes in the network as key gene targets. To further explore the mechanism of action of STZY in the treatment of RA, we divided the IL family genes involved into the pro-inflammatory and anti-inflammatory factor networks. Then, we used the molecular operating environment (MOE) software to perform molecular docking of the 2 types of immune network targets with their targeted compounds and active control drugs, and calculated the free binding energy. These experiments evaluated key protein targets, potential target compounds, and positive control drugs, and the 3D structures of the key protein targets IL-1*α*, IL-1*β*, IL-2, interleukin 4 (IL-4), IL-6, and interleukin 10 (IL-10) were downloaded from the RCSB Protein Data Bank (https://www.rcsb.org). The structures of these target protein were optimized with PyMOL (1.8) and MOE, and the 3D structures of the active compounds targeting the key protein was downloaded from the PubChem database (https://pubchem.ncbi.nlm.nih.gov). Compounds were imported into MOE for molecular docking and visual display.

In addition, in order to have a deeper understanding of the chemical and physical properties of the screened natural active compounds, we imported the SMILE numbers of the compounds into the SWISS ADME platform, and selected 5 parameters such as Water Solubility, GI absorption, Blood Brain Barrier, CYP2D6 inhibitor, and Lipinski as the screening directions for the prediction of their draggability.

## 3. Results

### 3.1. Channel tropism, active ingredients, and target genes of STZY

**Figure 1.**
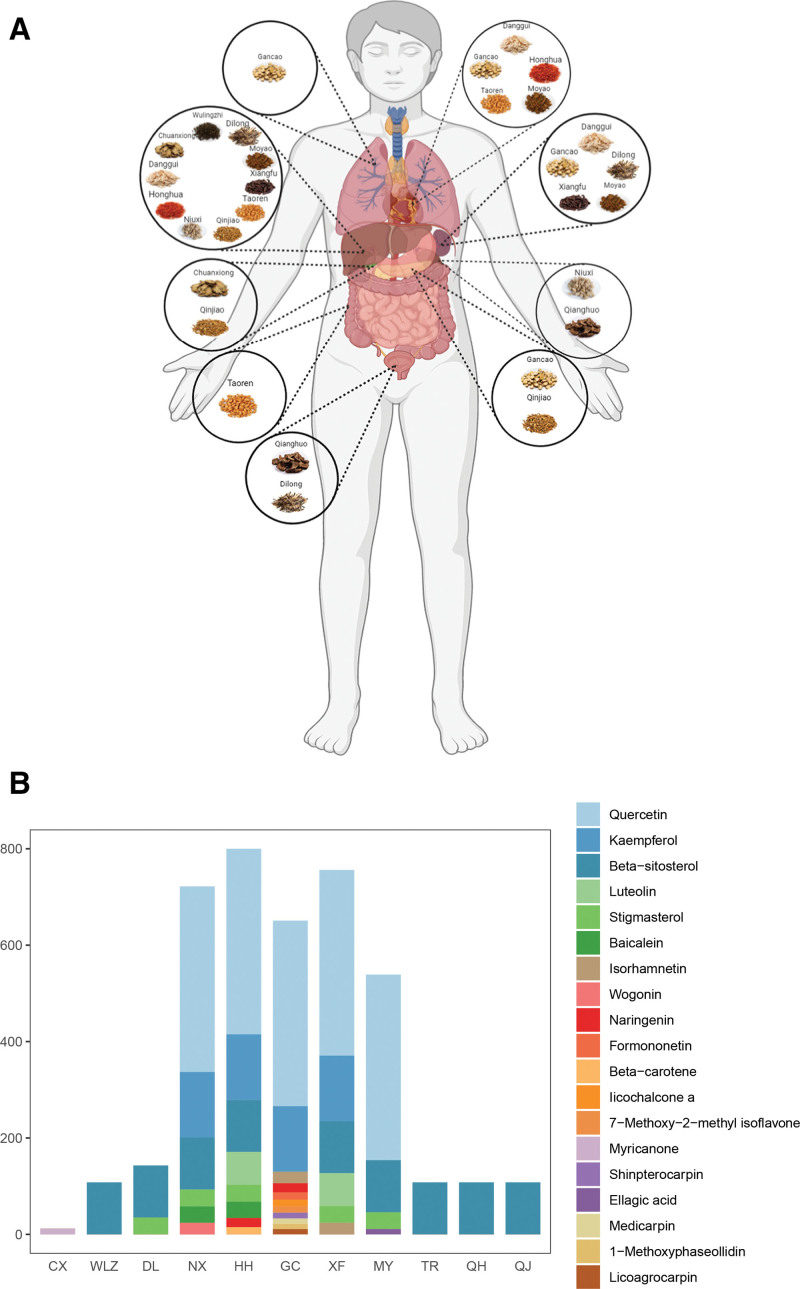
STZY channel tropism and potential active compounds in STZY. (A) The channel tropism of STZY. (B) Each rectangle corresponds to a numeric value that represents the number of genes identified for each TCM and potential compound. CX: Sichuan lovage rhizome, WLZ: flying squirrel droppings, DL: earthworm, NX: two toothed achyranthes root, HH: safflower, GC: licorice root, XF: nutgrass galingale rhizome, MY: myrrh, TR: peach seed, QH: incised notopterygium rhizome and root, QJ: largeleaf gentian root. STZY = *Shentong Zhuyu* decoction, TCM = Traditional Chinese Medicine.

STZY can be used to treat RA, and we aimed to determine the biological mechanisms of STZY in RA therapy. We investigated the herbs in the formula, namely *Angelicae Sinensis Radix* (Dang Gui), *Carthami Flos* (Hong Hua), *Persicae Semen* (Tao Ren), *Faeces Trogopterpri* (Wu Ling Zhi), *Achyranthis Bidentatae Radix* (Niu Xi), *Pheretima* (DI Long), *Chuanxiong Rhizoma* (Chuan Xiong), *Myrrha* (Mo Yao), *Cyperi Rhizoma* (Xiang Fu), *Gentianae Macrophyllae Radix* (Qin Jiao), *Notopterygii Rhizoma Et Radix* (Qiang Huo), and *Glycyrrhizae Radix Et Rhizome* (Gan Cao). Each herb has different properties, channel tropism, and flavor. The channel tropism for each of the ingredients in STZY was analyzed using the TCMIP database, which revealed that 10 of the ingredients were assigned to the liver meridian, 5 to the heart meridian, and 5 to the spleen meridian. Based on the TCM principle that 5 components in STZY have “warm properties,” the formulation can be considered a “warm medicine,” which primarily promotes blood circulation, resolves blood stasis, relieves pain, expels wind, and dehumidifies through the liver, heart, and spleen meridians (Fig. 1A). Based on Traditional Chinese Medicine Systems Pharmacology Database and Analysis Platform, TCMIP, and literature search, 256 active chemical compounds and 3988 potential targets were identified in STZY (Table 1). Posteriorly, we analyzed the frequency of the occurrence within the combined formula; quercetin, kaempferol, *β*-sitosterol, luteolin, stigmasterol, wogonin, naringenin, formononetin presented high occurrence, and were identified as potential active components in STZY (Fig. 1B).

**Table 1:** The components, properties, and channel tropism of *Shentong Zhuyu.*

Components	Property and flavor	Channel tropism
*Chuanxiong Rhizoma* (Chuan Xiong)	Warm, pungent	Gallbladder, liver
*Angelicae Sinensis Radix* (Dang Gui)	Warm, sweet, pungent	Liver, spleen, heart
*Glycyrrhizae Radix Et Rhizome* (Gan Cao)	Natured, sweet	Heart, lung, spleen, stomach
*Carthami Flos* (Hong Hua)	Warm, pungent	Liver, heart
*Achyranthis Bidentatae Radix* (Niu Xi)	Sweet, bitter, sour	Liver, kidney
*Notopterygii Rhizoma Et Radix* (Qiang Huo)	Warm, bitter, pungent	Bladder, kidney
*Gentianae Macrophyllae Radix* (Qin Jiao)	Bitter, pungent	Gallbladder, liver, stomach
*Persicae Semen* (Tao Ren)	Sweet, bitter	Large intestine, liver, heart
*Cyperi Rhizoma* (Xiang Fu)	Mildly sweet, Mildly bitter, pungent	Liver, spleen, triple burner
*Myrrha* (Mo Yao)	Bitter, pungent	Liver, spleen, heart
*Pheretima* (DI Long)	Salt, cold	Liver, spleen, bladder
*Faeces Trogopterpri* (Wu Ling Zhi)	Bitter and sweet, warm	liver

### 3.2. Acquisition of RA-related targets

We searched the GeneCards, DrugBank, online mendelian inheritance in man, therapeutic target database and PharmGKB databases using the keyword “Rheumatoid Arthritis” and obtained 1291 RA-related genes. Among these, 116 intersected with the STZY target genes and were identified as potential genes regulated by STZY in the treatment of RA (Figure S1, Supplemental Digital Content, http://links.lww.com/MD/K886).

### 3.3. “Active ingredient-target gene” network of STZY in RA treatment

**Figure 2.**
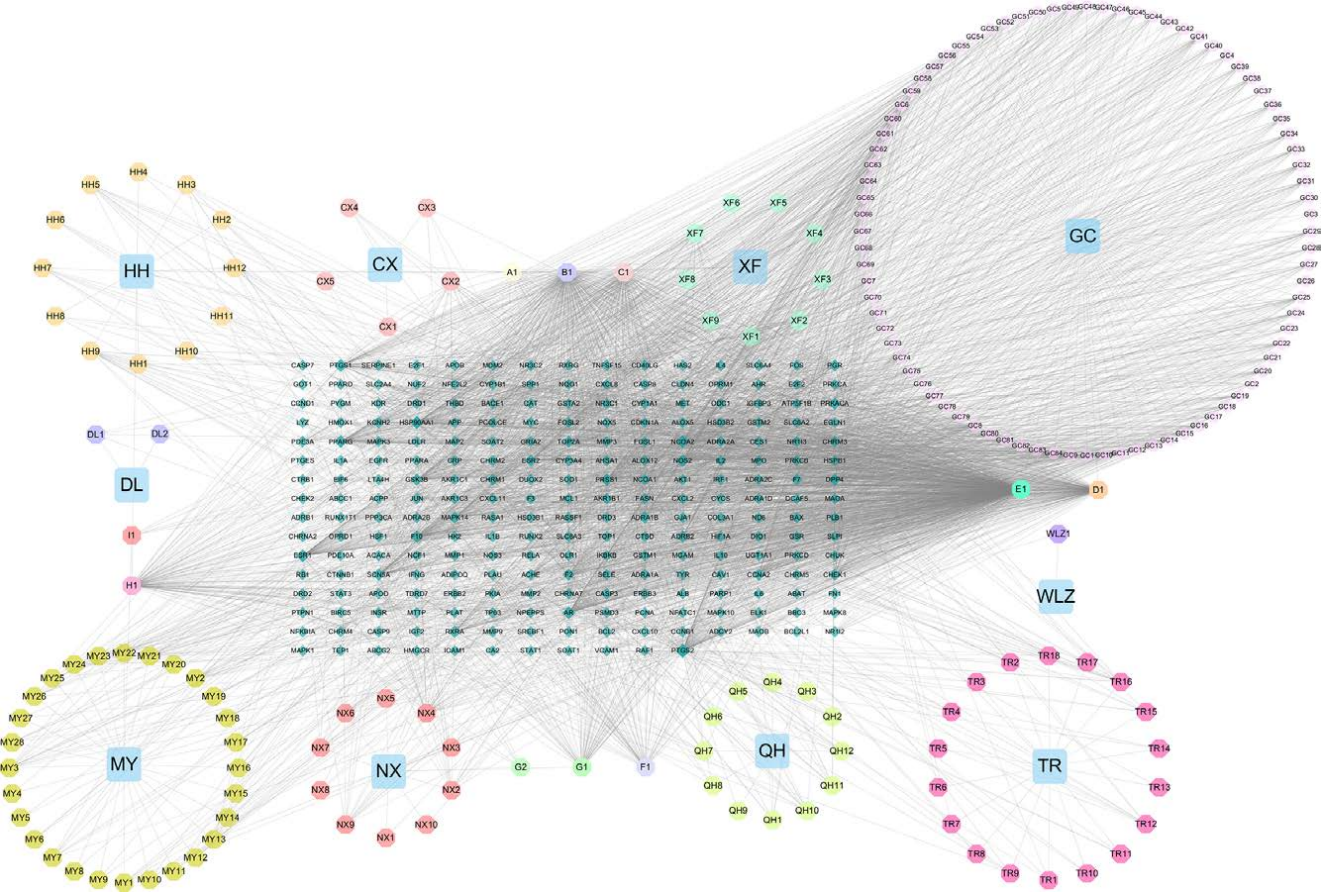
The “active substance-target gene” network and key core modules of STZY-RA. The octagon represents the active ingredient of the drug, the diamond represents the target gene, the rectangle represents the TCM, and the area represents the magnitude of the frequency value. CX1-5: Szechwan Lovage Rhizome-specific compounds, DL1-2: Earthworm-specific compounds, GC1-84: Licorice Root-specific compounds, HH1-12: Safflower-specific compounds, MY1-28: Myrrh-specific compounds, NX1-10: Common Achyranthes-specific compounds, QH1-12: Incised Notopterygium Rhizome- and Root-specific compounds, TR1-18: Peach Seed-specific compounds, and XF1-9: Nutgrass Galingale Rhizome-specific compounds. A1, B1, C1, D1, E1, F1, G1, G2, H1, and I1: compounds common to the formula. RA = rheumatoid arthritis, STZY = *Shentong Zhuyu* decoction, TCM = Traditional Chinese Medicine.

Based on the previous findings, we constructed the “formula-active ingredient-RA target genes” network using Cytoscape 3.7.2 to analyze the molecular mechanisms of STZY in RA treatment (Fig. 2), and used this network to identify the relationships between active compounds and their RA target genes (Table S1, Supplemental Digital Content, http://links.lww.com/MD/K887). Based on the results of network visualization, we hypothesized that quercetin, *β* sitosterol, kaempferol, stigmasterol, and luteolin are potential STZY compounds for RA treatment.

### 3.4. Enrichment analysis of targets of STZY in RA treatment

**Figure 3.**
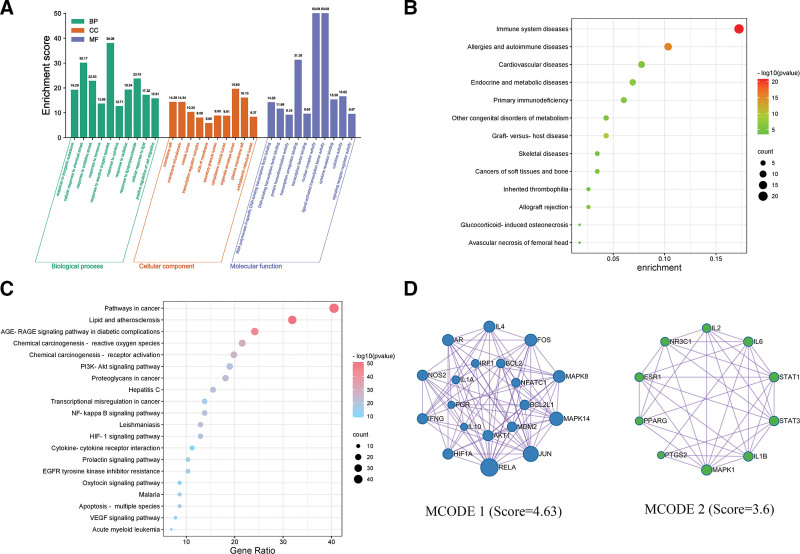
Enrichment analysis sets. (A) GO enrichment of RA samples treated with STZY. (B) disease enrichment evaluating STZY-RA intersectional genes. (C) Major signaling pathways identified in these interactions as represented by bubble plots. The number of genes enriched in each pathway is indicated by the size of the bubble. -Log10 *P* values are shown from small to large and from blue to red. The horizontal coordinate describes the proportion of genes in this pathway relative to the total number of genes. (D) MCODE network of key genes. GO = gene ontology, MCODE = molecular complex detection, RA = rheumatoid arthritis, STZY = *Shentong Zhuyu* decoction.

We analyzed the biological processes and signaling pathways affected by STZY. We used GO, KEGG, and DO enrichment to identify the target genes of the formula in RA treatment. The GO enrichment results indicated that the 116 target genes might have roles in biological processes, primarily associated with response to oxidative stress and chemical stress; cellular components, were primarily associated with membrane micro-regions, vesicular lumen, and membrane rafts; and molecular function, were mainly associated with DNA-binding transcription factor binding, protein homo-dimerization activity, and RNA polymerase II-specific DNA-binding transcription factor binding (Fig. 3A). Thus, we hypothesized that IL-1*α*, IL-1*β*, IL-2, IL-4, IL-6, and IL-10 may play critical roles in STZY-mediated treatment of RA.

Finally, DO enrichment analysis evidenced that immune system diseases, allergic and autoimmune diseases, graft-versus-host disease and primary immunodeficiency were significantly enriched (Fig. 3B). The top 20 signaling pathways of KEGG enrichment analysis suggested that the formula components are associated with reactive oxygen species, PI3K/AKT signaling pathway, nuclear factor kappa B (NF-κB) signaling pathway, cytokine-cytokine receptor interaction, apoptosis and hypoxia inducible factor 1 (HIF-1) signaling pathway (Fig. 3C). These results indicate that STZY largely exerts its anti-RA therapeutic effects through regulating signaling pathways such as PI3K/AKT, NF-κB, and HIF-1, via its effect on IL-1*α*, IL-1*β*, IL-2, IL-6, and IL-10. In addition, STZY might exerts its anti-RA therapeutic effects via regulating immunity, apoptosis and oxidative stress.

### 3.5. Network pharmacology analysis of “immune network targets-chemical compounds” interactions

**Figure 4.**
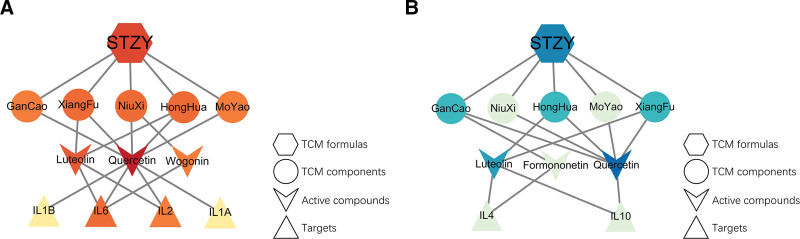
(A) “Formula - active compounds- pro-inflammatory factors” network. (B) “Formula - active compounds- anti-inflammatory factors” network. The diamond represents the TCM components, the triangle represents the active compounds, and the fusiform represents the network targets. Light to dark shading within the same category represents the increasing importance in balancing the “pro-inflammatory” effect. TCM = Traditional Chinese Medicine.

RA is a chronic autoimmune disease resulting from increased inflammation and dysregulated by inflammatory factors.^[19]^ The inflammatory factors participate in the progression of RA in different ways and are potential targets for the treatment of RA. Based on their function, inflammatory factors are classified as pro-inflammatory and anti-inflammatory. We used Metascape to analyze the key protein modules in the network, ultimately identifying 2 protein modules (score > 3.5) likely to be important in STZY-mediated treatment of RA (Fig. 3D), MCODE 1 is associated with inflammation, apoptosis, and cell migration, and MCODE 2 is associated with inflammation, apoptosis, and osteoporosis. These findings suggest that STZY may treat RA regulating inflammation, apoptosis, and osteoporosis. The key protein results of MCODE algorithm indicated that the pro-inflammatory factors IL-1α, IL-1*β*, IL-2, and IL-6 and the anti-inflammatory factors IL-4 and IL-10 may be the key immune network targets of RA, and the active compounds in STZY may play a role in treating RA by regulating the balance of immune network targets. Then we constructed the “formula-active compounds-pro-inflammatory factors” and “formula-active compounds-anti-inflammatory factors” networks to analyze the regulatory relationship between inflammatory factors and active compounds. The results indicated that the active compounds quercetin and luteolin from *Glycyrrhizae Radix et Rhizome* (Gan Cao), *Cyperi Rhizoma* (Xiang Fu), *Achyranthis Bidentatae Radix* (Niu Xi), *Carthami Flos* (Hong Hua), and *Myrrha* (Mo Yao) target a network of pro-inflammatory factors and regulate IL-1*α*, IL-1*β*, IL-2, and IL-6 expressions (Fig. 4A). Similarly, the active compounds quercetin, luteolin, and formononetin from the above Chinese herbal medicines target the anti-inflammatory factors network and regulate the expression of IL-4 and IL-10 (Fig. 4B). This results indicate that STZY might treat RA by balancing the pro-inflammatory and anti-inflammatory factors.

### 3.6. Validating “immune network target-compound” interactions through draggability prediction

**Figure 5.**
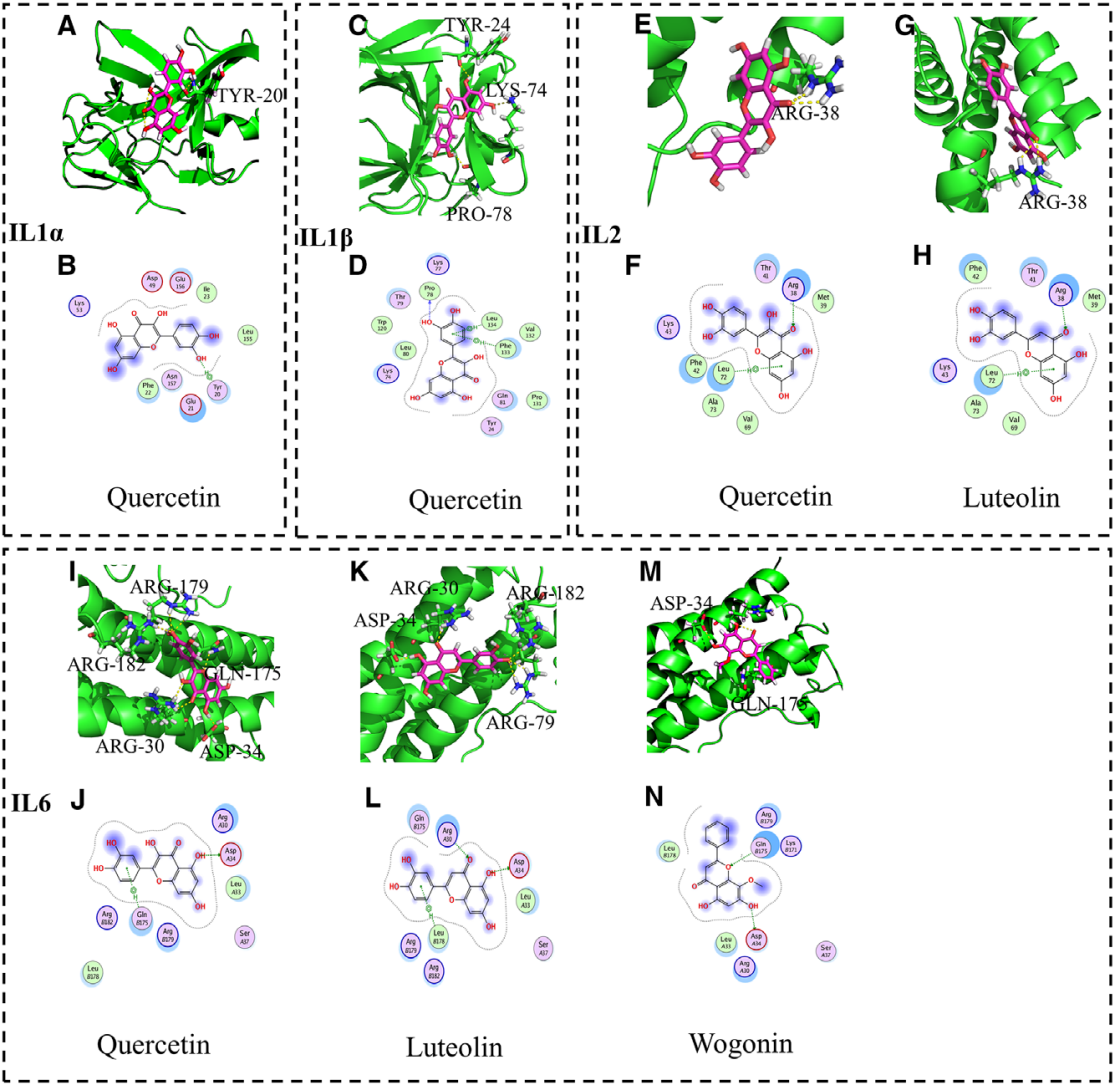
Binding patterns of pro-inflammatory factor networks and active compounds in STZY-mediated RA therapy. Binding patterns of (A) quercetin and IL-1*α*, (C) quercetin and IL-1*β*, (E) quercetin and IL-2, (G) luteolin and IL-2, (I) quercetin and IL-6, (K) luteolin and IL-6, (M) wogonin and IL-6. In (B), (D), (F), (H), (J), (L), and (N), the blue and green dashed lines indicate hydrogen bonds between the active compound and the main and side chain residues of the target, respectively. IL-1α = interleukin 1 alpha, IL-1β = interleukin 1 beta, IL-2 = interleukin 2, IL-6 = interleukin 6, RA = rheumatoid arthritis, STZY = *Shentong Zhuyu* decoction.

**Figure 6.**
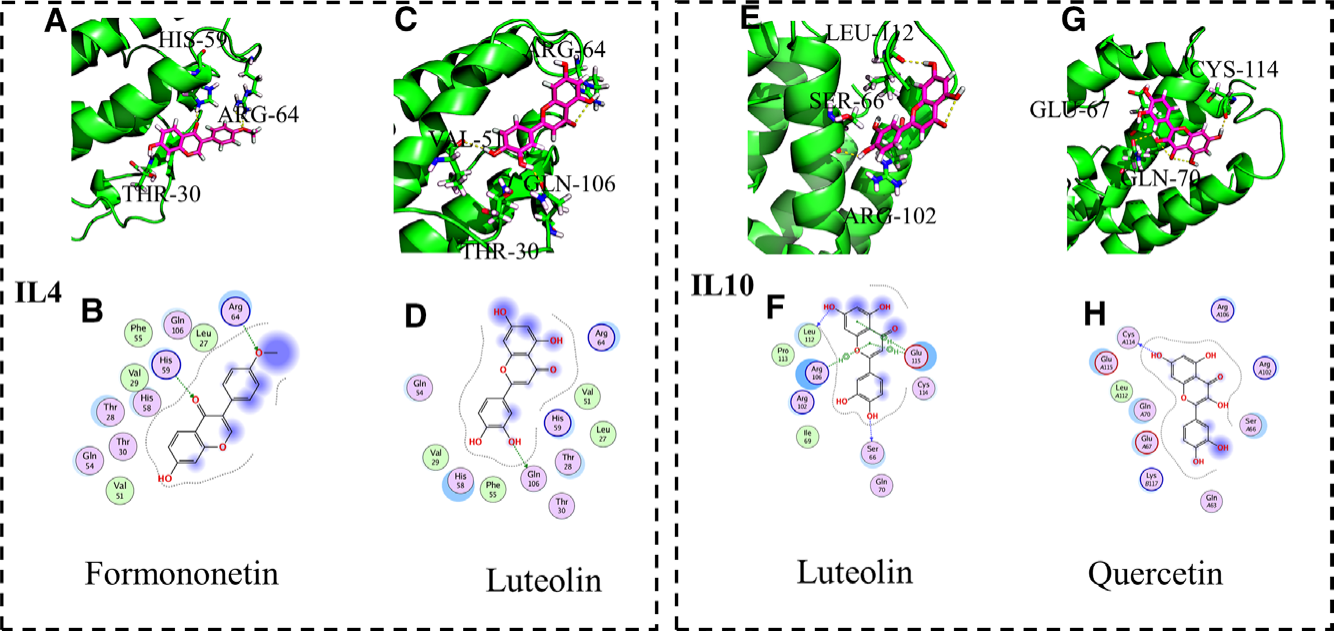
Binding patterns of key target proteins and active compounds in STZY-mediated treatment of RA. Binding patterns of (A) formononetin and IL-4, (C) luteolin and IL-4, (E) luteolin and IL-10 (G) quercetin and IL-10. In (B), (D), (F), and (H), the blue and green dashed lines indicate hydrogen bonds between the active compound and the main and side chain residues of the target, respectively. IL-4 = interleukin 4, IL-10 = interleukin 10, RA = rheumatoid arthritis, STZY = *Shentong Zhuyu* decoction.

To further verify whether STZY exerts its therapeutic effect on RA by regulating the pro-inflammatory and anti-inflammatory factors, we performed molecular docking to analyze the binding free energy between inflammatory targets and the active compounds obtained from the “immune network targets-chemical compounds” network. Binding free energy can be used to assess the stability of the target protein and active compounds; lower binding energy means more stable binding of the active compounds to the targets. Subsequently, we used MOE software to calculate the binding free energy of key targets IL-1*α*, IL-1*β*, IL-2, IL-6, and IL-10 to their target compounds, thereby assessing the stability of their molecular docking. The docking results evidenced that several active compounds could dock into the active pocket of immune targets (Figs. 5A–N, 6A–H).

The potential compounds quercetin and luteolin in STZY, as well as IL-6, IL-2, IL-1*β*, and IL-1*α* in the pro-inflammatory factor network, demonstrated binding activities that were nearly equivalent to or surpassed the binding activity of the active control drugs (leflunomide, kinofen, and hydroxychloroquine) when the molecule was docked (Table S2, Supplemental Digital Content, http://links.lww.com/MD/K888). Subsequently, we analyzed the docking results of the anti-inflammatory factor network, wherein the potential compounds quercetin, luteolin and formononetin in STZY evidenced binding activity similar to that of hydroxychloroquine and better binding activity than that of leflunomide when the molecules were docked.

We found that the binding free energy during IL-6 docking was the most stable within the pro-inflammatory factor network, while the binding free energy during IL-4 docking within the anti-inflammatory factor network was relatively stable. Therefore, we further analyzed these 2 scenarios. The assays outcomes revealed that, when docked with IL-6, quercetin exhibited better binding activity than kinofen, leflunomide and hydroxychloroquine, possibly because it can form hydrogen bonds with the ARG-179, GLN-175, ARG-182, ARG-30, and ASP-34 residues of IL-6 (Fig. 5I, G). Similarly, luteolin and wogonin exhibited better binding activity than kinofen and leflunomide when docked with IL-6. This finding may be attributed to the formation of hydrogen bonds between these compounds and the ARG-79, ARG-30, ARG-182, ASP-34 (Fig. 5K, L) and ASP-34 and GLN-175 (Fig. 5M, N) residues of IL-6.

Similarly, luteolin exhibited better binding activity than leflunomide when docked with IL-4, and had similar binding activity to those of kinofen and hydroxychloroquine. Based on the docking results, this enhancement may be due to the formation of hydrogen bonds with ARG-64, VAL-51, GLN-106, and THR-30 residues (Fig. 6C, D). Additionally, hydrogen bonds formed between formononetin and HIS-59, ARG-64, and THR-30 residues of IL-4, evidenced similar results to those of luteolin (Fig. 6A, B).

Meanwhile, the predicted results of the compounds druggability (Table S3, Supplemental Digital Content, http://links.lww.com/MD/K889) indicated that all 4 compounds were CYP2D6 inhibitory, had good aqueous solubility and gastrointestinal absorption, conformed to lipinski rule, and thus possessed the chemical and physical properties to become orally active drugs.

## 4. Discussion

RA has an incidence rate between 0.5% and 1.0% worldwide, and approximately 0.4% in China. To date, there are approximately 3 to 4 million patients with RA in China, predominantly young women, making RA one of the most significant causes of disability due to loss of functional capacity. No optimal treatment plan for this disease is available, and long-term medication is needed to control the condition. However, the long-term use of NSAIDs and glucocorticoids can increase the burden on the digestive tract, kidneys, and the cardiovascular system, leading to infections, osteoporosis, aseptic necrosis of the femoral head, and peptic ulcers. Moreover, some biological agents increase the risk of tumors. In addition, although various biological agents can achieve good treatment results in the short term, they are extremely expensive, ranging from several thousand to several tens of thousands yuan per month, resulting in prohibitive costs for most families. However, TCM offers an alternative; in TCM, RA is considered a type of Bi syndrome (arthralgia), which can be treated by resolving blood stasis and obstruction. Given that various pharmacological studies have evidenced that the active compounds in STZY act synergistically to support improved circulation, reduce inflammation, and promote the resolution of blood stasis, many believe that this medicine is an ideal option for treating RA.

Therefore, we used the active compounds from STZY, namely quercetin, kaempferol, β-sitosterol, luteolin, stigmasterol, wogonin, naringenin, and formononetin to identify the potential molecular targets of these compounds, and then evaluated their overlap with disease-specific targets in RA. Quercetin is known to stimulate the immune system, demonstrating its inhibition of the inflammatory response through the suppression of the NF-κB pathway and the reduction of pro-inflammatory cytokine IL-1*β* levels.^[20,21]^ Recent research demonstrated that quercetin can significantly reduce the transcription levels of multiple inflammatory cytokines, such as IL-1*α*.^[22]^ It has been demonstrated that quercetin can produce anti-inflammatory effects by inhibiting the secretion of the pro-inflammatory cytokine IL-6s^[23]^ while promoting the secretion of anti-inflammatory cytokine IL-10.^[24]^ In addition, quercetin can inhibit cytokines, such as VEGF and MMP-2, angiogenesis, and the formation of synovial scab to a certain extent. Quercetin plays a role in reducing inflammation and preventing the development of RA, therefore, we hypothesized that it can be a compound for the adjuvant treatment of RA.^[25]^ Suleimanov, TA et al^[26]^ confirmed the existence of quercetin and luteolin in *Carthami Flos* by synthesizing various methods. Kilani–Jaziri S et al^[27]^ confirmed the presence of the chemical compounds luteolin and quercetin in *Cyperi Rhizoma*. Kaempferol exerts various anti-inflammatory effects by inhibiting the expression of the pro-inflammatory factor IL-6,^[28,29]^ while *β*-sitosterol has a strong immunomodulatory function facilitated via reducing the expression of IL-2 and modulating NF-κB, a key molecule in inflammatory signaling.^[30,31]^ Stigmasterol significantly inhibits IL-1*β*-induced NF-κB signaling and suppresses cartilage degradation, suggesting that stigmasterol may inhibit osteoarthritis-induced cartilage degeneration.^[32,33]^ IL-1*β* and IL-6 levels in circulating blood were significantly reduced in mice with liver injury treated with wogonin, indicating that wogonin can inhibit the expression of pro-inflammatory cytokines during the inflammatory response.^[34]^ In in vitro tumor studies, wogonin may inhibit the binding activity of NF-κB and affect the transcription and expression of IL-6.^[35]^ Formononetin can be used for a variety of inflammatory-based diseases as an alternative source of classical drugs to prevent their evolution.^[36]^ Formononetin can inhibit the expression of pro-inflammatory cytokine IL-1*β*^[37]^ and play an anti-inflammatory role. Formononetin improved the innate immunity of experimental mice,^[38]^ formononetin may play an inflammatory role through the NF-κB signaling pathway.^[39]^ Fukai T et al^[40]^ used TLC and HPLC to determine the main active ingredients in *Glycyrrhizae Radix Et Rhizome*, of which formononetin is the main ingredient. Taken together, we can conclude that the primary active ingredients in STZY are likely to modulate RA via their anti-inflammatory effects, and that these effects are likely to be focused on the modulation of various inflammatory factors such as IL-1*β*, IL-1*α*, IL-2, IL-4, IL-6, and IL-10.

A large number of pro-inflammatory factors are needed to support the pathology of RA. The activation of PI3K/AKT signaling pathway is required to promote the pathological proliferation of fibroblast-like synoviocytes and facilitate pathogenesis. This scenario leads to the production of additional pro-inflammatory factors, creating a constant positive feedback loop, reenforcing their proliferation, and thus progressing the disease.^[41–43]^ Moreover, activated PI3K induces the expression of related factors and promotes pathological proliferation of osteoclasts (OC), eventually leading to bone destruction.^[44]^ The concurrent NF-κB activation leads to T cell activation, which induces increased inflammation and pathological fibroblast-like synoviocyte proliferation, stimulating OC proliferation and activation, leading to further bone destruction^[45]^ and resulting in a highly reenforcing pathological system. In addition to these effects, various immune mediators such as IL-6 are also upregulated via this positive feedback loop, adding an additional layer of support to the system.^[46]^ HIF-1*α* in the HIF-1 signaling pathway is also upregulated in response to NF-κB and an increase in hypoxia serves to support this hyperactivated immune environment, contributing to even more aggressive pathogenesis in the affected tissues.^[47]^ In addition, HIF-1*α* upregulates the expression of cytokines such as IL-1*β*^[48]^ and IL-6,^[49]^ which in turn increase the stability of the HIF-1*α* protein via their interactions with the reactive oxygen species pathway, which ultimately amplifies the inflammatory response.^[50]^ HIF-1*α* also plays an important role in the induction of bone destruction in RA via its inhibition of both osteogenic marker expression and osteoblast (OB) activity, thereby disrupting OB-OC homeostasis.^[48]^

Based on this evidence, we used network pharmacology and computer-aided drug design to investigate the molecular mechanisms underlying the STZY-mediated treatment of RA. This study included evaluation of various components, targets, and pathways, and explored the potential interactions of specific compounds during the treatment of RA. Our results demonstrated that quercetin and luteolin from *Achyranthis Bidentatae Radix, Carthami Flos, Glycyrrhizae Radix Et Rhizome, Cyperi Rhizoma, Myrrha* were the main active compounds in the STZY decoction, and that these compounds may exert various comprehensive osteoprotective effects via targeting of IL-1*α*, IL-1*β*, IL-2, IL-6, and IL-10 and subsequent inhibition of the PI3K/AKT, NF-κB, and HIF-1 pathways. We also suggest that these interactions help inhibit the inflammatory response and OC formation and promote an anti-inflammatory environment, reducing the clinical symptoms of RA. However, despite these crucial results, our study has some limitations. These include the limited number of papers describing the animal drug components in the formula and a lack of complete information on the screened active compounds and targets, meaning that some of the effects of this decoction remain unknown. In addition, these findings need to be validated in vitro and in vivo using metabolomics and proteomics approaches, and should be carefully evaluated for unwanted side effects. Nevertheless, this study provides the theoretical foundation for the development of new TCM-based drugs for the treatment of RA and promotes the use of active compound screening for target identification. In addition, we believe that some of the identified compounds may be further modified and optimized to produce improved drug candidates for the treatment of RA in the future.

## 5. Conclusion

Based on the results obtained using systems biology methods and review of existing research, we hypothesize that the STZY formulation mainly treats RA in two ways. On the 1 hand, it balances the immune network through active compounds. Luteolin, quercetin, wogonin, and other compounds act on pro-inflammatory cytokines, and luteolin, quercetin, and formononetin act on anti-inflammatory cytokines, thereby regulating the balance of immune networks. On the other hand, STZY also regulates the OB-OC balance and prevents bone destruction. In conclusion, the STZY compound can be used to treat RA by regulating immune network and OB-OC balances.

## Author contributions

**Conceptualization:** Shujun Bai, Xue Han, Yanchen Lan, Haodong Wang, Rui Wang, Liyuan Li.

**Formal analysis:** Shujun Bai, Xue Han, Yanchen Lan.

**Funding acquisition:** Qiuhang Song, Aiying Li.

**Software:** Shujun Bai, Xue Han.

**Supervision:** Qiuhang Song, Aiying Li.

**Validation:** Yanchen Lan, Haodong Wang, Rui Wang, Liyuan Li.

**Visualization:** Shujun Bai, Xue Han, Yanchen Lan.

**Writing – original draft:** Shujun Bai, Xue Han, Haodong Wang, Qiuhang Song.

**Writing – review & editing:** Yanchen Lan, Rui Wang, Qiuhang Song.

## Supplementary Material
