# Supplementary figures and images for: Mechanisms of action underlying Shentong Zhuyu decoction based treatment of rheumatoid arthritis using systems biology and computer-aided drug design
Source: Medicine (Baltimore). 2023 Nov 24;102(47):e36287. doi: 10.1097/MD.0000000000036287 (PMC10681588; doi:10.1097/MD.0000000000036287)

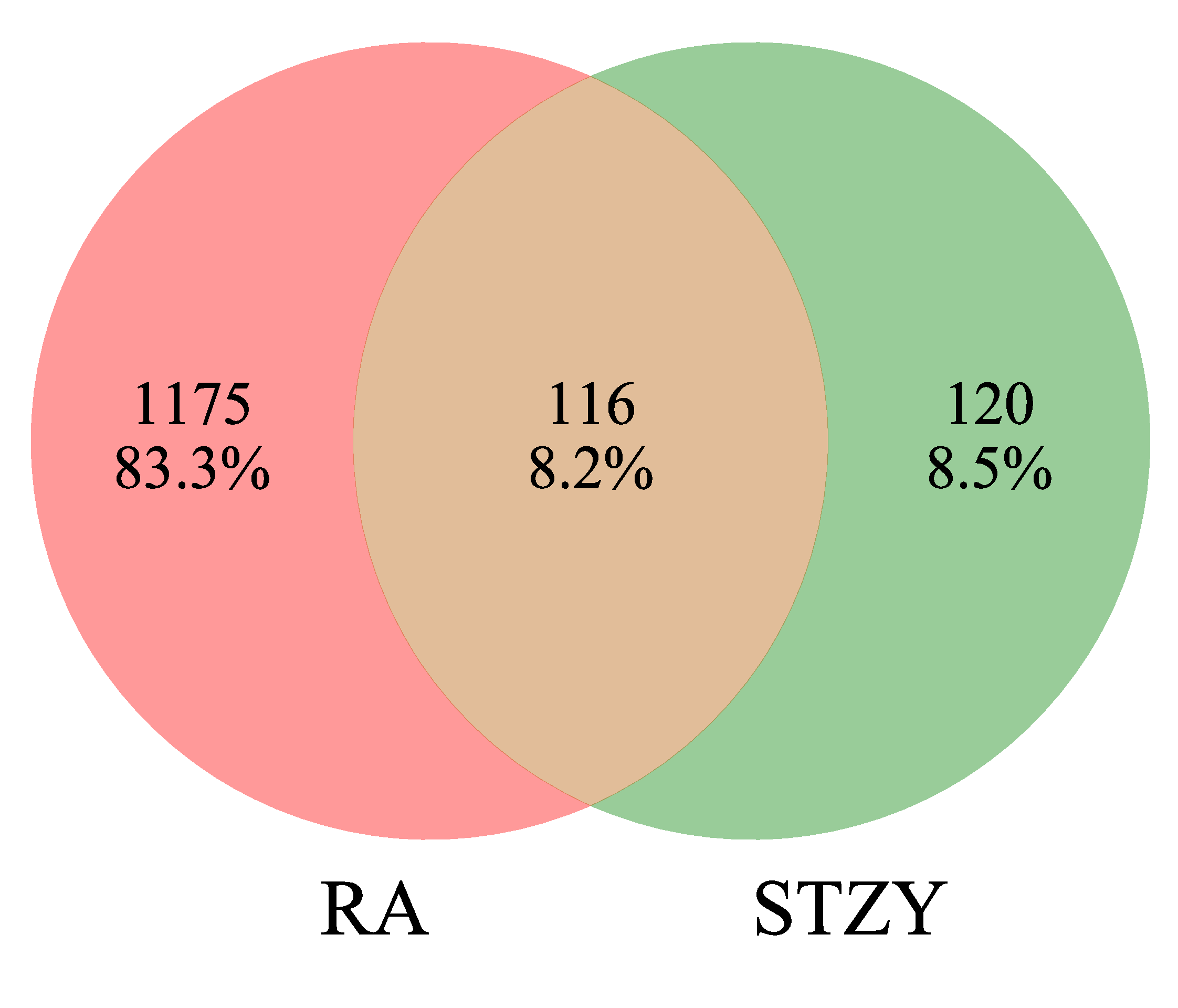

Supplement: Supplementary file 1 [file medi-102-e36287-s001.tif]
