# Supplementary material for: Mechanisms of action underlying Shentong Zhuyu decoction based treatment of rheumatoid arthritis using systems biology and computer-aided drug design
Source: Medicine (Baltimore). 2023 Nov 24;102(47):e36287. doi: 10.1097/MD.0000000000036287 (PMC10681588; doi:10.1097/MD.0000000000036287)
Supplement: Supplementary file 2 [file medi-102-e36287-s002.docx]

Supplementary Tables 2. Statistics of molecular docking of key targets of STZY with targeted compounds and clinical treatment of RA drugs

| Key targets | PDB ID | Combine free energy (kJ •mol^-1^) | | | | | | | | |
| --- | --- | --- | --- | --- | --- | --- | --- | --- | --- | --- |
|  |  | Quercetin | Luteolin | Formononetin | Wogonin | methotrexate | Kinnofen | Leflunomide | Sulfasalazine | Hydroxychloroquine |
| IL-6 | 1ALU | -5.3808 | -5.1765 | — | -5.1449 | -6.0665 | -4.8852 | -4.8344 | -5.9086 | -5.2692 |
| IL-2 | 1M48 | -5.4960 | -5.4238 | — | — | -7.4010 | -5.5483 | -5.5147 | -6.8895 | -5.8014 |
| IL-2 | 1PW6 | -5.0286 | -5.4128 | — | — | -6.9044 | -5.8552 | -5.3777 | -6.4587 | -5.7287 |
| IL-2 | 1PY2 | -5.4315 | -5.3850 | — | — | -6.7948 | -5.7241 | -5.3241 | -6.5126 | -5.8604 |
| IL-1*β* | 6Y8M | -4.5782 | — | — | — | -6.3409 | -4.9601 | -4.7942 | -6.0195 | -5.4320 |
| IL-1*β* | 5R8Q | -5.3670 | — | — | — | -6.3939 | -6.5471 | -5.0363 | -5.9917 | -5.7159 |
| IL-1*α* | 2KKI | -5.2172 | — | — | — | -6.1575 | -5.4350 | -5.0324 | -5.5750 | -5.5691 |
| IL-4 | 2D48 | — | -5.4019 | -5.2041 | — | -6.3356 | -5.4193 | -5.1991 | -5.7801 | -5.4225 |
| IL-10 | 1LK3 | -5.5900 | -5.5102 | — | — | -6.9273 | -6.1610 | -5.2772 | -6.3942 | -5.8515 |

PDB ID = RCSB Protein Data Bank ID, IL-6 = Interleukin-6, IL-2 = Interleukin 2, IL-1*β* = Interleukin 1 Beta, IL-1α= Interleukin 1 Alpha, IL-4 = Interleukin 4, IL-10 = Interleukin 10
