# Supplementary material for: Mechanisms of action underlying Shentong Zhuyu decoction based treatment of rheumatoid arthritis using systems biology and computer-aided drug design
Source: Medicine (Baltimore). 2023 Nov 24;102(47):e36287. doi: 10.1097/MD.0000000000036287 (PMC10681588; doi:10.1097/MD.0000000000036287)
Supplement: Supplementary file 4 [file medi-102-e36287-s004.docx]

Supplementary Table 3 Predictive results of the pharmacogenetic properties of the formula STZY

| Compound Name | Water Solubility | GI absorption | Blood Brain Barrier | CYP2D6 inhibitor | Lipinski |
| --- | --- | --- | --- | --- | --- |
| quercetin | Soluble | High | No | Yes | Yes |
| luteolin | Soluble | High | No | Yes | Yes |
| formononetin | Moderately soluble | High | Yes | Yes | Yes |
